# A systematic review and meta-analysis of the direct epidemiological and economic effects of seasonal influenza vaccination on healthcare workers

**DOI:** 10.1371/journal.pone.0198685

**Published:** 2018-06-07

**Authors:** Chisato Imai, Michiko Toizumi, Lisa Hall, Stephen Lambert, Kate Halton, Katharina Merollini

**Affiliations:** 1 Centre for Health Systems and Safety Research, Australian Institute of Health Innovation, Macquarie University, Sydney, New South Wales, Australia; 2 Department of Pediatric Infectious Diseases, Institute of Tropical Medicine, Nagasaki University, Nagasaki, Japan; 3 Epidemiology and Biostatistics Division, School of Public Health, Faculty of Medicine, The University of Queensland, Herston, Queensland, Australia; 4 Institute of Health and Biomedical Innovation, Queensland University of Technology, Brisbane, Queensland, Australia; 5 UQ Child Health Research Centre, School of Medicine, The University of Queensland, Brisbane, Queensland, Australia; 6 Faculty of Science, Health, Education and Engineering, University of the Sunshine Coast, Sunshine Coast, Queensland, Australia; University of Washington, UNITED STATES

## Abstract

**Background:**

Influenza vaccination is a commonly used intervention to prevent influenza infection in healthcare workers (HCWs) and onward transmission to other staff and patients. We undertook a systematic review to synthesize the latest evidence of the direct epidemiological and economic effectiveness of seasonal influenza vaccination among HCW.

**Methods:**

We conducted a systematic search of MEDLINE/PubMed, Scopus, and Cochrane Central Register of Controlled Trials from 1980 through January 2018. All studies comparing vaccinated and non-vaccinated (i.e. placebo or non-intervention) groups of HCWs were included. Research articles that focused on only patient-related outcomes or monovalent A(H1N1)pdm09 vaccines were excluded. Two reviewers independently selected articles and extracted data. Pooled-analyses were conducted on morbidity outcomes including laboratory-confirmed influenza, influenza-like illnesses (ILI), and absenteeism. Economic studies were summarized for the characteristics of methods and findings.

**Results:**

Thirteen articles met eligibility criteria: three articles were randomized controlled studies and ten were cohort studies. Pooled results showed a significant effect on laboratory-confirmed influenza incidence but not ILI. While the overall incidence of absenteeism was not changed by vaccine, ILI absenteeism was significantly reduced. The duration of absenteeism was also shortened by vaccination. All published economic evaluations consistently found that the immunization of HCW was cost saving based on crude estimates of avoided absenteeism by vaccination. No studies, however, comprehensively evaluated both health outcomes and costs of vaccination programs to examine cost-effectiveness.

**Discussion:**

Our findings reinforced the influenza vaccine effects in reducing infection incidence and length of absenteeism. A better understanding of the incidence of absenteeism and comprehensive economic program evaluations are required to ensure the best possible management of ill HCWs and the investment in HCW immunization in increasingly constrained financial environments. These steps are fundamental to establish sustainability and cost-effectiveness of vaccination programs and underpin HCW immunization policy.

## Introduction

Influenza is a highly contagious viral illness that poses a considerable hazard in healthcare facilities. The clinical attack rate of influenza among inpatients can be much higher than among the general population due to the vulnerability of hospital patients with underlying conditions [1], and consequently nosocomial outbreaks are frequently reported [2–4]. From an economic perspective, nosocomial influenza infections lead to excess healthcare costs due to additional clinical complications among infected patients, and the loss of productivity and replacement of staff among ill HCWs [5, 6].

Seasonal influenza vaccination of HCWs is a pivotal measure for prevention and control efforts in healthcare settings. International guidelines stress the significance of this approach with recommendations of organizational strategies to immunize HCW annually [7, 8]. However, the challenge in maintaining high vaccination coverage of HCW has been marked by unfavorably low compliance rates [9, 10], and limited improvement by voluntary motivations through incentives and provision of free vaccine [11, 12]. The use of mandatory or regulatory policy has consequently gained popularity and has been implemented in organizations based on the premise of indirect patient benefits [13, 14], while the evidence for this as a primary driver for HCW programs remains contentious [15].

Given the uncertainty in attributions of patient benefits to HCW vaccination, having strong evidence of the direct effectiveness of vaccination on healthcare workers and the cost-effectiveness of these campaigns in reducing the incidence of illness and absenteeism among HCW is important. Previously, a systematic review evaluated the direct effectiveness of influenza vaccination of HCWs but failed to provide any conclusions due to the limited number of included studies [16]. Moreover, only epidemiological effects were examined, and no systematic review has summarized economic evidence despite the substantial costs involved in implementing HCW vaccination.

We conducted a systematic review with expanded selection criteria to include more relevant articles than the previous review and also considered both epidemiological and economic effectiveness. The specific objective in this review was to synthesize evidence to whether influenza vaccines reduced influenza related morbidity among HCWs, which includes incidence rate and absenteeism, and the associated costs of these programs.

## Materials and methods

We followed the Preferred Reporting Items for Systematic Reviews and Meta-Analyses (PRISMA) Statement for reporting all outcomes of our systematic review (S1 File).

### Selection criteria

This review included both randomized clinical trials (RCT) and comparative observational studies (e.g. cohort and case-control studies) that compared the effects of influenza vaccination on HCWs with placebo or no intervention. HCW—our study population—were defined as employees in any healthcare facilities who could be exposed to patients directly or indirectly. This included both clinical staff (e.g. doctors, nurses, allied health professionals) and administrative officers. The intervention of interest was seasonal influenza vaccination, which is generally administered prior to the expected epidemic season. Vaccines, live attenuated or inactivated virus, by any route were considered.

Studies that focused on non-seasonal influenza or specifically with monovalent A(H1N1)pdm09 vaccines that were produced as a response to the 2009 global influenza pandemic were excluded. As the focus of this work was specific to HCW endpoints, any articles that investigated vaccine effects solely on patient-related outcomes from vaccinating either patients or HCWs were also excluded.

We assessed two primary outcomes of interest: health and economic effects of influenza vaccination. Health outcomes included the incidence of influenza infections (laboratory-confirmed or clinically-diagnosed only), and the incidence and length of absenteeism after vaccination. The key economic outcome was cost-effectiveness that compares the relative cost and health outcomes of HCW vaccination.

### Search strategy

The studies included in this systematic review were collected from electronic searches of the PubMed/MEDLINE, Scopus and Cochrane Central Register of Controlled Trials from 1980 to January 2018. The search was conducted with combinations of the following terms: (influenza) AND (vaccin* OR immunization OR immunisation) AND (healthcare OR health care OR hospital OR clinic* OR medical OR nurs* OR physician OR doctor). The details of the search strategies are presented in the supplementary material (S1 Table). For additional specification of studies, the medical literature was restricted to journal articles written in English using the filter functions of languages and publication types on the electronic databases.

### Selection process and data extraction

Two reviewers (CI & MT) independently screened the titles and abstracts of the articles identified by the electronic searches, and selected articles for full-text reading in accordance with the eligibility criteria. The full-texts for the relevant articles were then retrieved and independently evaluated further by the reviewers to determine inclusion. Reference lists of included articles were also screened to consider further potentially eligible trials. The discrepancies in study selection were resolved by discussion between the reviewers until a consensus was reached.

Data extraction was performed systematically using a form designed in accordance with the Cochrane Handbook for Systematic Reviews of Interventions [17]. We extracted the following data: description of setting, study method, characteristics of participants, description of vaccine, outcomes, and methodological quality. The data were entered into Review Manager (RevMan) version 5.3 software [18].

### Assessment of risk of bias

The quality assessment of included articles was independently carried out by two review authors (CI & MT). Disagreements were resolved by discussion between the two assessing authors. We used the Cochrane’s tool [17] and the Newcastle-Ottawa Scale (NOS) [19] to evaluate the risk of bias for RCTs and observational studies, respectively. The Cochrane tool requires evaluations of six domains: selection bias, performance bias, detection bias, attrition bias, reporting bias, and other sources of bias. Each RCT study was classified into unclear, low, or high risk for each domain. The NOS uses the star system with a maximum nine stars and evaluates three domains with a total eight items (S2 File): the selection of the study groups, the comparability of the groups, and the ascertainment of outcome of interest. Each item of NOS was rated by unclear (no star), low risk (one or more stars), or high risk (no star). We further categorized the quality of observational studies as follows: seven to nine stars was labelled high quality, five to six stars moderate quality, and four or fewer stars low quality.

### Summary of measures and synthesis

Meta-analyses were performed on the extracted epidemiological data using Review Manager (RevMan) version 5.3 [18] to evaluate for the summary measures. We calculated a pooled risk ratios (RR) and 95% confidence intervals (CI) for dichotomous outcome variables, which included the incidence of clinically-diagnosed influenza (influenza-like illness; ILI), laboratory-confirmed influenza, and absenteeism. An RR indicates the ratio of an event probability in a vaccinated group compared to an unvaccinated group. An RR less than 1 equates to influenza vaccine providing a protective effect against infections or sick leave. For continuous parameters such as the lengths of absenteeism, we calculated the mean differences with 95% CI between the two groups. In order to account for the clinical and methodological diversity within the included articles, random effects models with the inverse variance (IV) were applied in computing the summary measures of vaccine effects in meta-analyses.

Heterogeneity among the studies was evaluated using Q statistic-based *χ*^2^ test and *I*^2^ statistic. P-values less than 0.1 for the *χ*^2^ test were considered significantly heterogeneous. We used *I*^2^ values to interpret the degrees of heterogeneity by using the Cochrane guideline [17]: <30% little concern; 30% to 75% moderate heterogeneity; >75% substantial heterogeneity. Outcomes reported by only one study and articles with missing standard errors were not included in meta-analyses.

In synthesizing economic outcomes, we did not conduct quantitative analyses due to the variations of the currencies and analysis methods across the studies. We instead carried out a narrative overview by characterizing the cost analyses in terms of cost elements taken into account and findings of whether influenza vaccines were cost-effective or not as the summary synthesis.

### Sensitivity and sub-group analyses

Given the potential biases underpinned by different study designs, we performed all syntheses of epidemiological results with subgroup analyses by study design (i.e. RCT vs. observational studies) as a priori defined groups. If there were heterogeneities within or between study designs, we further investigated with other potential factors as post-hoc analyses to identify what factors drove the heterogeneity.

To assess the robustness of our results, we carried out separate analyses with exclusions of observational studies graded as low quality (studies with 4 stars or fewer). If a study appeared to be an outlier, it was excluded from meta-analyses to assess variability in the overall estimate.

### Assessment of publication bias

Considering the limited number of the studies in each comparison, we could not construct funnel plots and calculate Egger’s test to investigate publication bias.

## Results

### Results of the search

**Fig 1:**
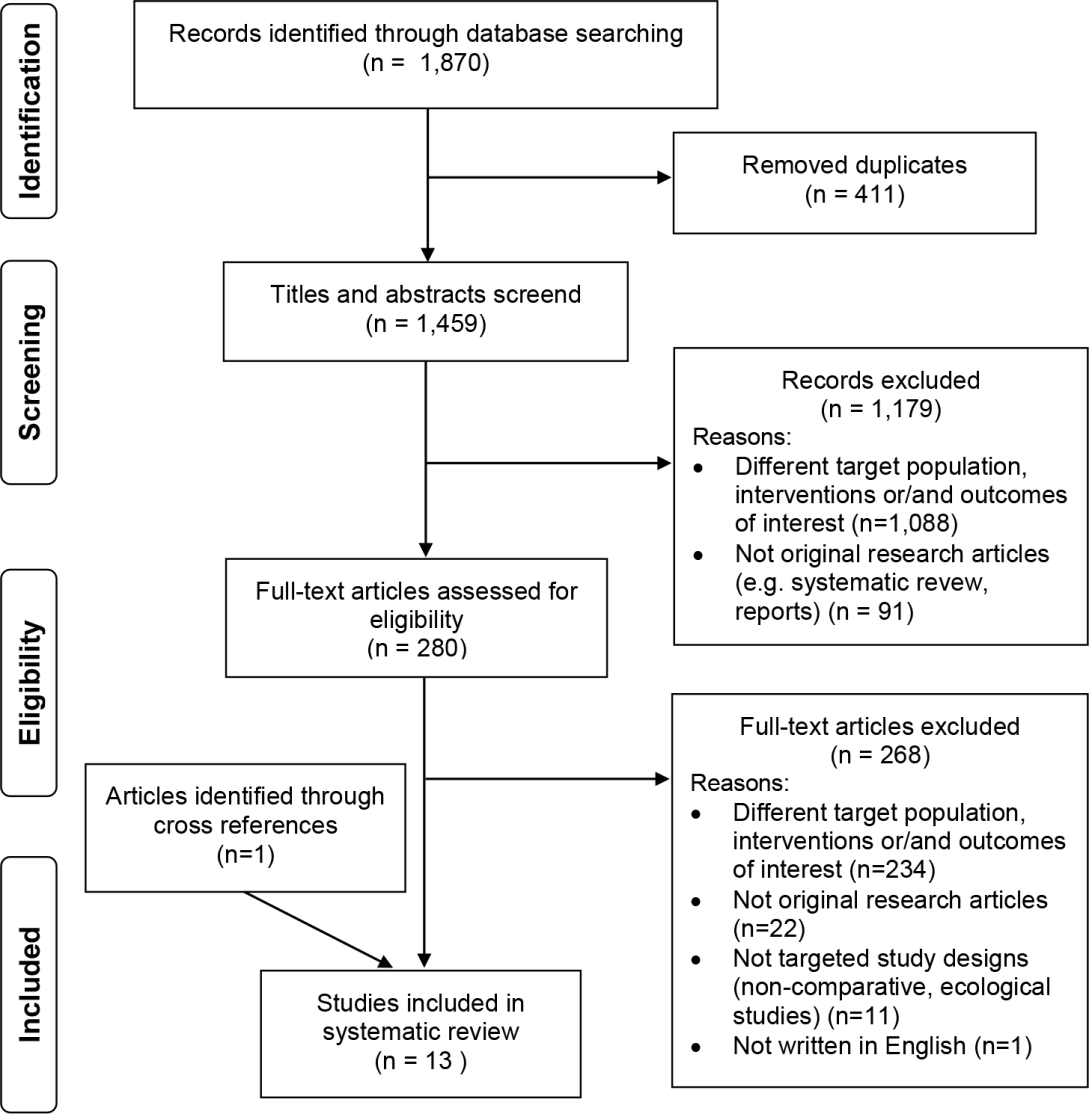
PRISMA flow diagram. The flow of study selection.

The initial search of the electronic databases identified 1,870 articles, of which we retrieved full-text of 280 articles after we removed duplicates, and screened titles and abstracts. The subsequent full-text review identified 12 articles as eligible studies with exclusions of 268 articles due to different study population, interventions, and outcomes of interest (e.g. behavioral studies), study design (e.g. ecological studies), or articles not written in English (Fig 1). We added a further article after reviewing the reference lists from the selected articles. Consequently, a total of 13 studies were included in our systematic review.

### Characteristics of the studies

The selected studies included three RCTs [20–22], two prospective cohort studies [23, 24], and eight retrospective cohort studies [25–32] (Table 1). There were 20,282 participants enrolled in the included studies, of which 5,083 participants received influenza vaccinations and 15,199 participants had no intervention or placebo. All studies, except one study exclusively focusing on economic evaluation [28], conducted epidemiological evaluations for health outcomes, five of which [21, 27, 30–32] also performed cost-benefit analyses based on estimated epidemiological effects. While one study targeted general practitioners in a study region, the remainder included hospital- or healthcare facility-based employees. Further detailed characteristics and outcomes of the included studies are provided (S2 and S3 Tables).

**Table 1:** Characteristics of included studies.

Study	Country	Study location	Study Design^a^	Participants	Sample size	
					Vaccinated	Control
Amadio (2010)	Italy	Hospital	RC	HCWs	215	2,393
Chan (2007)	Hong Kong	Emergency department	RC	Clinical staffs (no doctors)	33	40
Chan (2008)	Taiwan	Hospital	RC	HCWs	367	40
Colombo (2006)	Italy	Public healthcare facility	RC	HCWs	107	107
Ishikane (2016)	Japan	long-term care facility	RC	HCWs	288	50
Ito (2005)	Japan	Hospital	RC	HCWs	237	129
Kheok (2008)	Singapore	Hospital	PC	HCWs	211	330
Michiels (2006)	Belgium	Flanders region	PC	GPs	55 (2002) 30 (2003)	33(2002) 36(2003)
Saxén (1999)	Finland	Hospital	RCT	HCWs	216	211
Thomson (1999)	Australia	Hospital	RC	HCWs	748	3,844
Van Buynder, (2015)	Canada	Public healthcare facility	RC	HCWs	2,360	7,719
Weingarten (1988)	U.S.	Hospital	RCT	HCWs	91	88
Wilde (1999)	U.S.	Hospital	RCT	Doctors, nurses, respiratory therapists	180	179

^a^ PC, prospective cohort; RC, retrospective cohort; RCT, randomized controlled trial.

### Risk of bias in included studies

**Fig 2:**
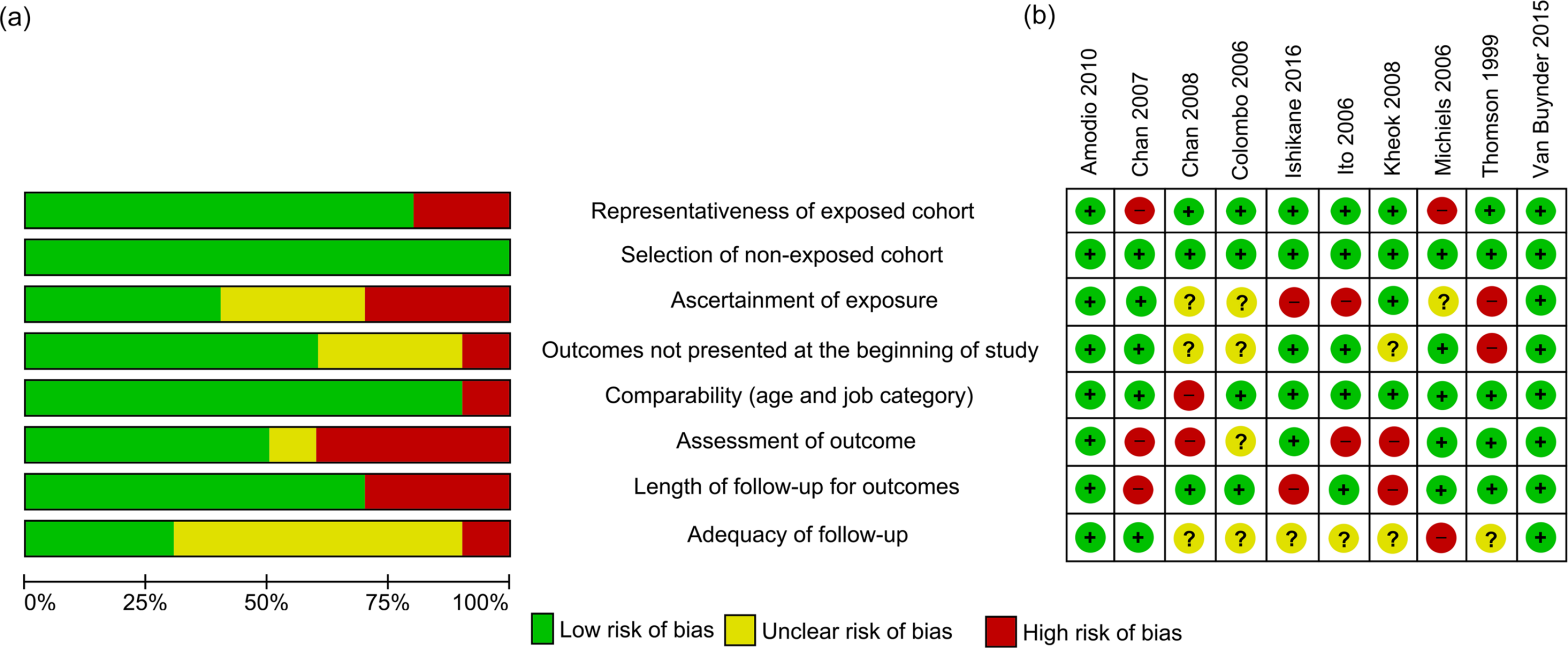
Assessment of risk of bias for observational studies. (a) Risk of bias graph showing each item presented as percentages across all observational studies (b) Risk of bias summary showing each item for each study.

Commonly identified risks of biases in observational studies were related to the validity of evidence in regard to exposure (i.e. vaccine uptake) and outcome variables. It is worth noting that self-reported data through questionnaires or surveys were commonly used in studies, and consequently the risks of bias in ascertainment of exposure and assessment of outcome were assessed as potentially high. There was also uncertain risk in adequacy of follow-up due to insufficient information in several observational studies (Fig 2).

The quality of the observational studies was moderate assessed: three studies were classified as high quality [25, 29, 32], five studies were moderate [24, 27, 28, 30, 31], and two studies were low quality [23, 26] (S4 Table).

**Fig 3:**
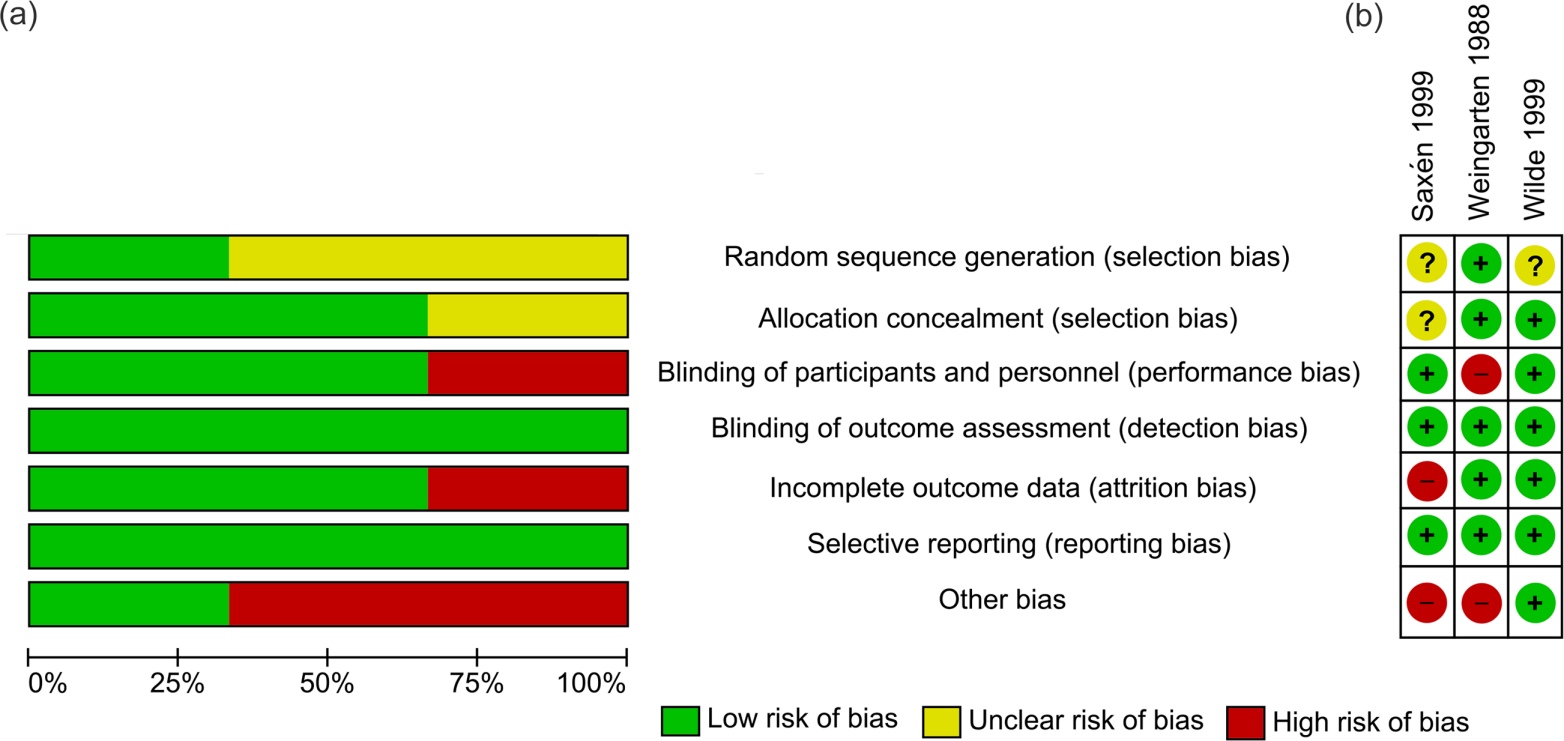
Assessment of risk of bias for RCTs. (a) Risk of bias graph showing each item presented as percentages across all RCT studies (b) Risk of bias summary showing each item for each study.

In the RCTs, the risk of selection bias was the most uncertain of all biases as there was a lack of information regarding randomization procedures in two of the three studies (Fig 3). There was one study each at high risk for performance and attrition bias. Other biases were considered at highest risk due to the potential of bias from self-reporting and differential recall in two studies.

### Influenza-like illness (ILI)

ILI is a clinical, non-laboratory-confirmed diagnosis of respiratory infection. The differences in ILI incidence rates between vaccinated and unvaccinated HCW groups were reported in four observational studies [23, 24, 27, 28] and one RCT [21]. In the five studies, a total of 1,578 HCWs were enrolled. The clinical definitions to diagnose ILI cases in the studies were more or less similar, requiring either or both of systemic (e.g. fever, chill, myalgia) and respiratory tract symptoms (e.g. rhinorrhea, sore throat, cough, hoarseness).

**Fig 4:**
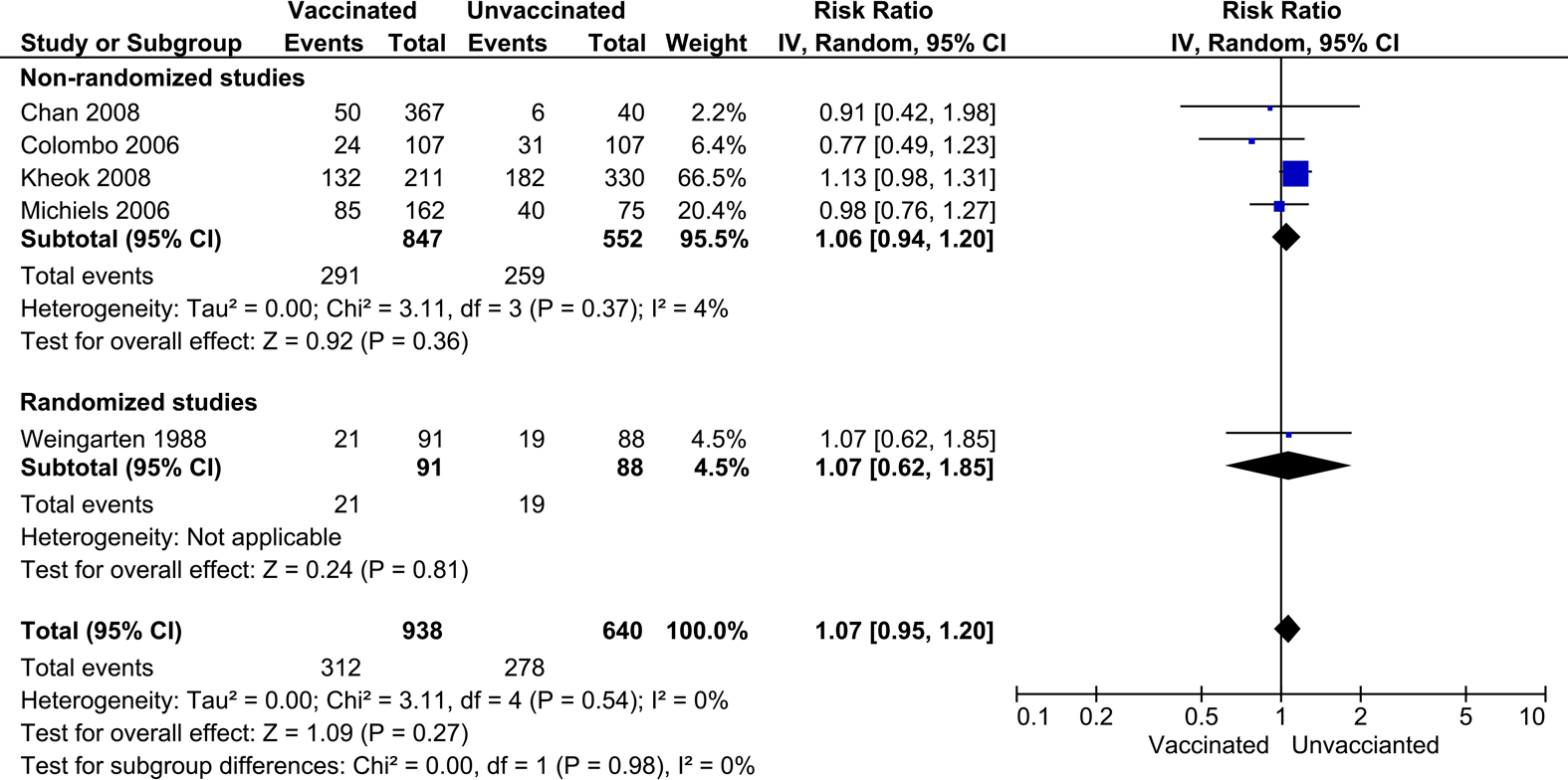
Forest plot showing the vaccine effects on ILI incidence. The effects of seasonal influenza vaccines on the ILI incidences between vaccinated and unvaccinated HCWs by study design.

Regardless of study design, whether observational study or RCT, there were no studies reporting a clear effect of influenza vaccines on ILI detection in HCW (Fig 4). Likewise, the pooled effect among the all five studies did not find a statistically significant effect (pooled RR = 1.07, 95%CI; 0.95–1.20). There were no significant heterogeneities existing within (observational studies; *I*^2^ = 4%, p = 0.37) and between study designs (*I*^2^ = 0%, p = 0.98).

#### Laboratory-confirmed influenza cases

There were three observational studies [24, 29, 30] and one RCT [22] using laboratory-confirmed influenza cases to assess the difference in the incidence rates between the vaccinated and unvaccinated groups. In the four studies, a total of 1,464 HCWs were enrolled. The pooled effect among the observational studies showed that the number of laboratory-confirmed cases among vaccinated group was significantly lower than unvaccinated group (pooled RR = 0.50, 95% CI; 0.33–0.76). The significant protective effect of the vaccine against influenza infection was similarly reported in the RCT as well (RR = 0.12, 95% CI; 0.04–0.41). The overall pooled RR across the all four studies was 0.40 (95% CI; 0.23–0.69) (S1 Fig).

**Fig 5:**
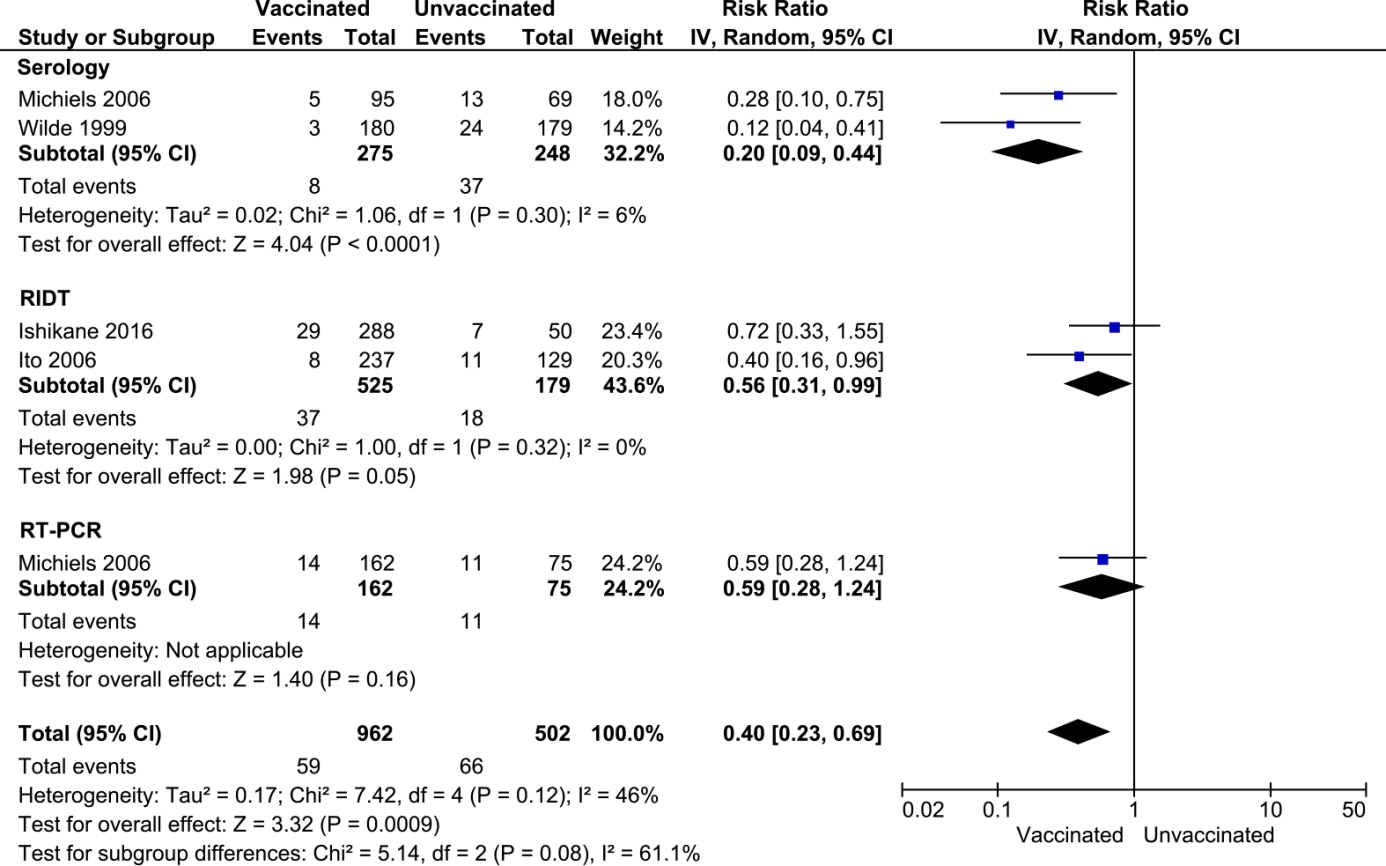
Forest plot showing the vaccine effects on laboratory-confirmed cases. The effects of seasonal influenza vaccines on the laboratory-confirmed cases between vaccinated and unvaccinated HCWs by laboratory method.

However, there was high heterogeneity in the measured effects between the RCT and a group of the observational studies (*I*^2^ = 79%, p = 0.03), while there was no significant heterogeneity among the observational studies (*I*^2^ = 0%, p = 0.45). To evaluate whether the heterogeneity could be attributed to the difference in the study designs, we further examined the heterogeneities among the groups by types of laboratory tests for varying levels of sensitivity and specificity, which included serology (one RCT [22] and one observational study [24]), rapid influenza diagnostic test (RIDT) (two observational studies [29, 30]), and reverse transcription-polymerase chain reaction (RT-PCR) groups (one observational study [24]) (Fig 5). In the updated subgroup analysis, we found no significant heterogeneity within group but a moderate heterogeneity existed between groups (*I*^2^ = 61%, p = 0.08). Two-pairs comparisons suggested the serology group as being a potential source of the heterogeneity in the results: there was no significant difference between two non-serology groups, but when each non-serology group was compared with the serology group they were heterogeneous (vs. RIDT: *I*^2^ = 76%, p = 0.04; vs. RT-PCR: *I*^2^ = 74.2%, p = 0.05). Serological testing showed a much stronger preventative effect of influenza vaccine (pooled RR = 0.20, 95% CI: 0.09–0.44) than RIDT (pooled RR = 0.56, 95% CI: 0.31–0.99) and RT-PCR (RR = 0.59, CI: 0.28–1.24).

### Absenteeism

Vaccine impacts on absenteeism were assessed by the examining the incidence and mean duration of absenteeism in the included studies. The causes of absenteeism varied by study, and included ILI, all-cause illness, and laboratory-confirmed influenza.

**Fig 6:**
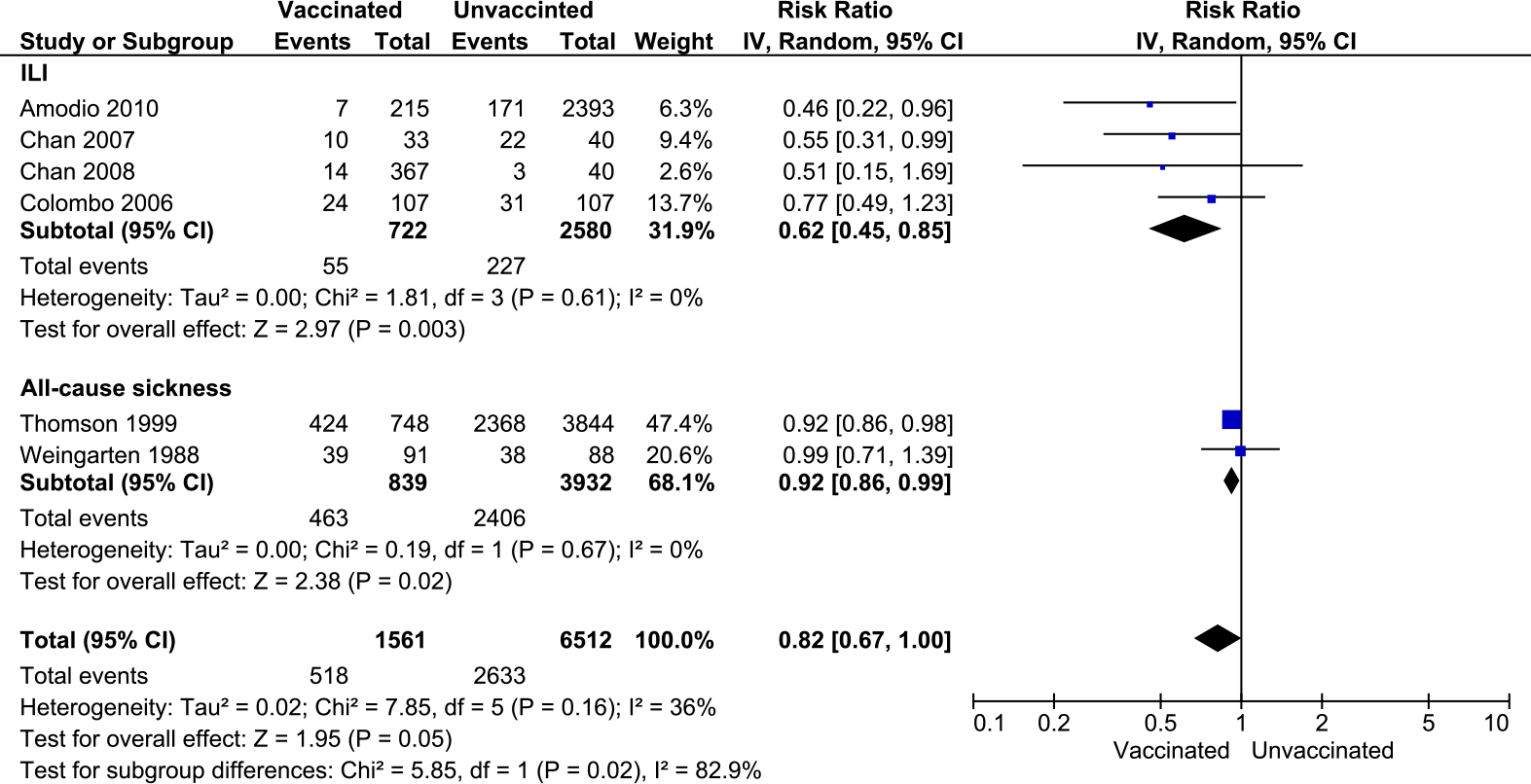
Forest plot showing the vaccine effects on absenteeism incidence. The effects of seasonal influenza vaccines on the absenteeism incidence between vaccinated and unvaccinated HCWs by causes of sick leave.

In the analyses using incidence data, there were five observational studies [25–28, 31] and one RCT [21] with a total enrollment of 8,073 HCWs. Before conducting a meta-analysis, the subgroup analysis by study design revealed no significant but a moderate level of heterogeneity both within observational studies (*I*^2^ = 47%, p = 0.11) and between study designs (*I*^2^ = 45%, p = 0.18)(S2 Fig). In order to ensure the potential source of heterogeneity, we attempted another sub-group analysis by causes of absenteeism, which included ILI and all-cause illness. The updated analysis with cause-specific subgroups then showed no significant heterogeneity within subgroup but a substantial heterogeneity between them (*I*^2^ = 82.9%, p = 0.02), which indicated the causes of absenteeism as an underlying source of heterogeneity (Fig 6). The meta-analysis showed influenza vaccine significantly reduced the incidence of absenteeism due to ILI (pooled RR = 0.62, 95%CI; 0.45–0.85). Influenza vaccination also reduced the incidence of absenteeism due to all-cause illness, however, the strength of the effect was less evident (pooled RR = 0.92, 95%CI = 0.67–0.99). Altogether, the pooled estimate of all six studies showed an ambiguous association between vaccination and absenteeism (overall pooled RR = 0.82, 95%CI = 0.67–1.00).

**Fig 7:**
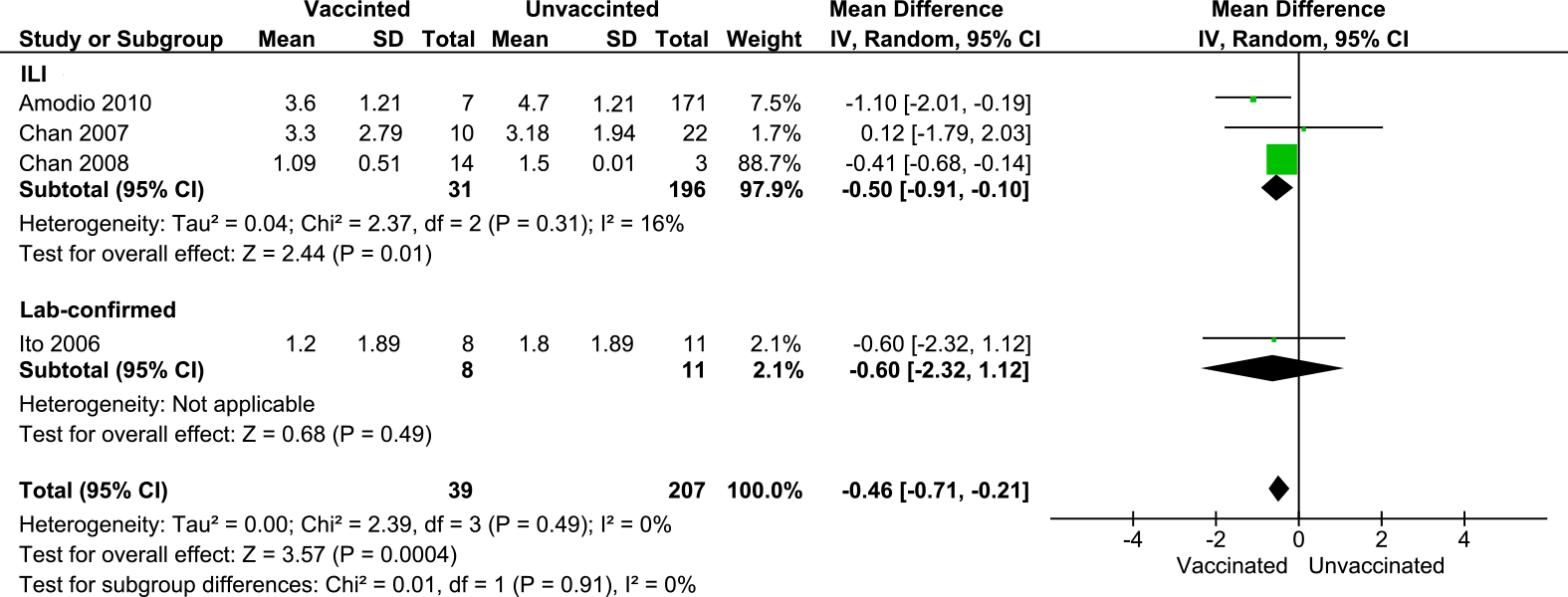
Forest plot showing the vaccine effects on absenteeism duration among ill HCWs. The effects of seasonal influenza vaccines on the mean days of sick leave between vaccinated and unvaccinated HCWs who took sick leave. Analyses were conducted by causes of sick leave.

There were four observational studies with analyses on the mean durations of absenteeism among those who took leave from work [25–27, 30]. There were 246 HCWs enrolled in the studies. The causes of absenteeism in the studies was ILI except for one study [30] which analyzed cases with laboratory-confirmed infections (Fig 7). In synthesizing the measured effects, if a study reported in hours, we recalculated to days with an assumption of eight hours for one working day. The pooled analyses showed that the difference between unvaccinated and vaccinated group in absenteeism due to ILI and laboratory-confirmed infections were -0.50 days (95%CI = -0.91 –-0.10) and -0.60 (95%CI = -2.32–1.12) respectively. There was no heterogeneity between the two groups with the different sick leave causes (*I*^2^ = 0%, p = 0.91). The overall pooled analysis estimated that a vaccinated group had a significantly shorter sick leave– 0.46 days shorter–than an unvaccinated group (95% CI; -0.71 –-0.21, p < 0.01).

### Sensitivity analysis

The overall effect after removing the low quality studies continued to show no significant difference in the ILI incidence between vaccinated and unvaccinated groups (S3 Fig). The sensitivity analysis for sick leave incidence did not noticeably alter the pooled effect of each subgroup (e.g. ILI and all-cause), but the heterogeneity between the two subgroups changed from significant to non-significant (*I*^2^ = 69.4%, p = 0.07). As the overall effect of sick leave incidence by vaccination, the sensitivity analysis added a clarity to the early inconclusive finding that there was no significant effect of the vaccine (pooled RR = 0.88, 95%CI = 0.75–1.03, p = 0.11) (S4 Fig). In the sensitivity analysis for the mean days of absenteeism, the pooled difference between vaccinated and unvaccinated groups within a subgroup of ILI shifted towards 0 and the vaccine effect became marginal (pooled difference = -0.61 days, 95%CI = -1.22–0.00, p = 0.05). However, as previously observed, there was no heterogeneity between the two cause-specific subgroups (i.e. ILI and laboratory-confirmed absenteeism) and the overall result remained to show the significant vaccine effect of reducing sick leave duration (S5 Fig). We did not conduct a sensitivity analysis for the incidence of laboratory-confirmed cases as there was no low-quality study to exclude.

### Economic effects

There were 6 studies that performed partial economic evaluations of influenza immunization among HCWs [21, 27, 28, 30–32]. There was no study that carried out a full cost-effectiveness assessment in which cost per health outcome (e.g. quality-adjusted life years: QALYs) was measured to compare the values of monetary costs and quality of life.

The general cost analyses in the included studies estimated the expenses of influenza vaccination to hospital or healthcare organizations from the employers’ perspective. Consequently, no consideration was given to “out-of-pocket” costs for HCWs, such as costs of doctor visits and medicines to treat influenza or ILI symptoms.

In all cost analyses, avoided hours of sick leave among vaccinated HCWs were considered as beneficial and cost-saving which overweighed the cost of program implementation. Their findings subsequently illustrated the evidence of gaining economic benefits through immunization programs, as they found vaccination reduced the incidence and duration of absenteeism regardless of the significance of the statistical association. Two of the studies [30, 32] reported the potential cost benefits based on the estimated avoided hours of sick leave in their studies. The other studies [21, 27, 28, 31] also emphasized the same interpretation of their results after taking into account other various cost components which included direct (i.e. vaccines, equipment, or time for vaccine administration) and indirect costs (i.e. working time lost for immunization) (Table 2).

**Table 2:** Cost elements for cost-benefit evaluations.

Cost items		Chan(08)	Colombo	Ito	Thomson	Van Buynder	Weingarten
**Direct cost**							
	Vaccines	(√)*	-	-	-	√	-
	Equipment (e.g. syringe, needle)	√	-	√	√	√	-
	Nurses’ time for vaccine administration	-	-	√	√	√	-
**Indirect cost**							
	Working time lost for immunization	-	-	√	-	√	√
	Working time lost due to adverse effects	-	√	√	√	√	√
	Doctor visits and medicine for adverse effects or ILIs	√	√	√	√	√	√

√, an element taken into account in cost-benefit evaluations.

*The cost was known but was not included in the calculation of the expenses to an employer as a local authority procured them.

## Discussion and conclusions

Our pooled analyses found a significant effect of seasonal influenza vaccination reducing influenza infection of HCW when defined using laboratory-confirmed cases, but not by ILI. This was not surprising considering the clinical influenza diagnosis has a limited positive predictive value for identifying influenza infection. During winter months when influenza epidemics occur, other respiratory pathogens, such as parainfluenza and respiratory syncytial viruses, that share the common medical symptoms of ILI are also circulating [33–35]. Laboratory testing results are more accurate diagnoses of influenza infections and is, therefore, likely to show a stronger preventative effect. It is worth noting, however, that the predictive probability of laboratory tests can also vary for several reasons such as the specific test characteristics, the timing of testing and collection of the samples, testing patterns (sicker patients getting tested), and the prevalence of circulating virus [36]. In our pooled analyses, serologically-defined cases showed a strong preventative effect against influenza infection while the vaccine effects of cases identified through other diagnostic tests (RIDT and RT-PCR) cases were not as evident. This heterogeneity was seemingly attributed to the methodological differences. The sensitivity of RIDT is approximately 50%–70% and reported to be lower than viral culture and RT-PCR which are considered reference standards of laboratory-confirmed influenza [36]. In terms of RT-PCR in our study, there was only one study included for review and consequently we could not conclusively evaluate the effect. Nonetheless, the overall vaccine effect on laboratory-confirmed cases (pooled RR = 0.40, 95% CI; 0.23–0.69) in our analysis is very close to the previous finding from a systematic review on influenza vaccine among healthy adults (pooled RR = 0.39, 95% CI:0.30–0.52) [37]

HCW influenza vaccination reduced the length of absenteeism due to ILI or laboratory-confirmed infections, consistent with the vaccine reducing the clinical severity of influenza. Understanding the findings regarding the incidence or number of staff who took sick leave, was somewhat more challenging because this finding was not consistent with our other findings relating to influenza incidence. Specifically, we did not observe a vaccine effect on overall incidence of absenteeism, while influenza infections confirmed by laboratory tests were reduced by vaccination. Although this could be simply explained that the cause of absenteeism was not due to laboratory-confirmed influenza, the discrepancy between the incidences of influenza infection and absenteeism also occurred even with a focus on ILI. Vaccination reduced the incidence of ILI absenteeism while a reduction in ILI incidence was not observed. This uncorrelated relationship between the two outcomes may appear to be contradictory, but may reflect the complexity in the association between vaccination and absenteeism where not all ill cases will take sick leave. The decision whether to take sick leave involves various individual factors such as the severity of symptoms, health awareness, work and home environment, and finances [38–40]. In view of our unique study population–HCWs, absenteeism particularly requires attention and careful evaluation as the culture of ill presenteeism (e.g. working while ill) among this specific population has been previously highlighted in recent literature [41–43]. We speculate that, while vaccination may have helped with preventing infection and reducing the symptomatic duration of influenza, various behavioral factors and attitudes toward sick leave could have influenced actual leave occurrence.

All economic evaluations investigated the costs based on the employers’ perspective, highly focusing on the prevention of work absenteeism as the main benefit of HCW immunization. Economic evaluations primarily demonstrated by how much monetary savings resulting from absenteeism overweighed the costs associated with a vaccination program. There was, however, variability in the type of cost input parameters included for analyses across reviewed studies, which makes comparisons difficult. The results of the cost evaluations in the reviewed articles were also not sufficient to determine whether HCW vaccination is indeed cost-effective in a given healthcare setting. In general, the inclusions of both cost and health outcomes are essential properties in performing a cost-effectiveness analysis. However, the reviewed studies did not consider all relevant monetary and non-monetary outcomes relevant to influenza vaccination; for instance, adverse effects related to vaccinations and costs of medical treatments for sick HCWs. Where possible, future studies should consider both health and cost estimates to evaluate cost-effectiveness, which provides decision-makers with a fuller picture of implications when implementing a vaccination program for HCWs.

In a previous systematic review, only RCT studies were included to evaluate effects of seasonal influenza vaccines among HCWs. As a result, the review included only three articles published more than fifteen years ago and were able to perform a pooled analysis on only one outcome (i.e. absenteeism) [16]. Considering the limited number of RCT studies, we expanded the selection criteria to include all best available evidence, aiming to increase the power to examine vaccine effects on HCWs. Although a combination of different study designs is less common for meta-analyses, it has been suggested that heterogeneity of studies can be beneficial by adding scientific values and clinical relevance of the results [44, 45]. In addition, we carefully performed the pooled analyses by separating the different study designs as subgroups and removing the observational studies of low quality for sensitivity analyses. Our results remained consistent with no distinct heterogeneity even after considering study designs or quality of study, which supports the validity of reporting the overall pooled effects across all included studies in our review.

There were some limitations in this study. First, the vaccines types stated in the studies were all trivalent inactivated, but the vaccines in four studies were unidentified. We assumed that the studies similarly used trivalent inactivated unless stated otherwise, because the vaccine was most common in the population of healthy adults and the studies were conducted before the new quadrivalent vaccine was first licensed and recommended its usage for seasonal immunization by WHO in 2013 [46]. Secondly, there may be a numerous factors driving the underlying differences in the included studies that subsequently lead to the variation of their reported outcomes. For instance, many studies have shown annual variations of influenza vaccine effects due to the differences of population demographics, matching rates between vaccine and circulating strains, epidemic intensity, and carry-over effects from previous immunization [47–49]. In HCW populations, job category (e.g. administration, medical, maintenance) and working unit (e.g. ER, pediatrics) have been also reported as possible effect modifiers and confounders for varying vulnerability and exposure to patients at high risk. Needless to say, the study populations in our reviewed studies were not identical and it is unknown to what extent the studies could have inherited these sources of heterogeneities. We, however, did observe neither apparent outliers nor significant heterogeneities in the studies that couldn’t be explained by other reasons such as study designs, types of laboratory test, or causes of absenteeism.

While there have been movements towards mandatory HCW immunization, the controversy over infringement of individual autonomy persistently remains with a lack of strong supporting evidence of the benefits of HCW vaccination [11, 50]. This systematic review provides important evidence for decision makers on the effectiveness of influenza vaccination in reducing incidence of influenza and absenteeism duration amongst HCW. We now need better evidence around absenteeism incidence and cost-effectiveness of HCW immunization to address these issues and better implement programs whilst considering ethical concerns.

## Supporting information

S1 File PRISMA checklist. (PDF)

S2 File Newcastle-Ottawa quality assessment scale for cohort studies. (PDF)

S1 Table The literature search strategy. (PDF)

S2 Table Characteristics of the included studies. (PDF)

S3 Table Epidemiological and economic outcomes. (PDF)

S4 Table The NOS score results of the observational studies. (PDF)

S1 Fig Forest plot showing the vaccine effects on laboratory-confirmed cases. The effects of seasonal influenza vaccines on the laboratory-confirmed cases between vaccinated and unvaccinated HCWs by study design.
(PDF)

S2 Fig Forest plot showing the vaccine effects on absenteeism incidence. The effects of seasonal influenza vaccines on the absenteeism incidence between vaccinated and unvaccinated HCWs by study design.
(PDF)

S3 Fig The sensitivity analysis for ILI incidence after removing studies with low quality. (PDF)

S4 Fig The sensitivity analysis for absenteeism incidence after removing studies with low quality. (PDF)

S5 Fig The sensitivity analysis for the mean days of absenteeism after removing studies with low quality. (PDF)

## Abbreviations

CI: Confidence interval
HCW: Healthcare worker
ILI: Influenza-like illness
IV: Inverse variance
NOS: Newcastle-Ottawa Scale
PRISMA: Preferred Reporting Items for Systematic Reviews and Meta-Analyses
QALY: Quality-adjusted life year
RCT: Randomized controlled trial
RIDT: Rapid influenza diagnostic test
RR: Risk ratio
RT-PCR: Reverse transcription-polymerase chain reaction
