# Supplementary material for: A systematic review and meta-analysis of the direct epidemiological and economic effects of seasonal influenza vaccination on healthcare workers
Source: PLoS One. 2018 Jun 7;13(6):e0198685. doi: 10.1371/journal.pone.0198685 (PMC5991711; doi:10.1371/journal.pone.0198685)
Supplement: S2 File — (PDF) [file pone.0198685.s002.pdf]

S2 File. Newcastle-Ottawa Quality Assessment Scale for cohort studies.

Note: A study can be awarded a maximum of one star for each numbered item within the Selection and Outcome categories. A maximum of two stars can be given for Comparability

Selection

1) Representativeness of the exposed cohort
   - a) truly representative of the average HCW in the community *****
   - b) somewhat representative of the average HCW in the community *****
   - c) selected group of users e.g. only nurses, volunteers
   - d) no description of the derivation of the cohort

2) Selection of the non-exposed cohort
   - a) drawn from the same community as the exposed cohort *****
   - b) drawn from a different source
   - c) no description of the derivation of the non-exposed cohort

3) Ascertainment of exposure
   - a) secure record (e.g. surgical records) *****
   - b) structured interview *****
   - c) written self-report
   - d) no description

4) Demonstration that outcome of interest was not present at start of study
   - a) yes *****
   - b) no

Comparability

1) Comparability of cohorts on the basis of the design or analysis
   - a) study controls for age or years of employment *****
   - b) study controls for job category, other demographic info, or health condition

Outcome

1) Assessment of outcome
   - a) independent blind assessment *****
   - b) record linkage *****
   - c) self-report
   - d) no description

2) Was follow-up long enough for outcomes to occur
   - a) yes *****
   - b) no

3) Adequacy of follow up of cohorts
   - a) complete follow up - all subjects accounted for *****
   - b) subjects lost to follow up unlikely to introduce bias - small number lost. ≥ 80% follow up, or description provided of those lost *****
   - c) follow up rate < 80 % and no description of those lost
   - d) no statement
