# Supplementary material for: A systematic review and meta-analysis of the direct epidemiological and economic effects of seasonal influenza vaccination on healthcare workers
Source: PLoS One. 2018 Jun 7;13(6):e0198685. doi: 10.1371/journal.pone.0198685 (PMC5991711; doi:10.1371/journal.pone.0198685)
Supplement: S1 Table — (PDF) [file pone.0198685.s003.pdf]

S1 Table. The literature search strategy

Database	Search strategy
Medline/PubMed<br>Scopus	influenza AND (vaccin* OR immunization OR immunisation) AND (healthcare OR health care OR hospital OR clnic* OR medical OR nurs* OR physician OR doctor [Title]
Cochrane library	#1 influenza [Title]<br>#2 vaccin* OR immunization OR immunisation [Title]<br>#3 healthcare OR health care OR hospital OR clnic* OR medical OR nurs* OR physician OR doctor [Title]<br>#4 #1 AND #2 AND #3
