# Supplementary material for: A systematic review and meta-analysis of the direct epidemiological and economic effects of seasonal influenza vaccination on healthcare workers
Source: PLoS One. 2018 Jun 7;13(6):e0198685. doi: 10.1371/journal.pone.0198685 (PMC5991711; doi:10.1371/journal.pone.0198685)
Supplement: S2 Table — (PDF) [file pone.0198685.s004.pdf]

S2 Table. Characteristics of the included studies

Study	Study location, period, research	Study design	Study participants	Primary outcomes	Influenza/case definition
Amadio (2010)	Italy, 2007-2008<br>A university hospital (Epi)	Retrospective cohort study<br>Vaccination: Unknown	All HCWs who worked full-time or part-time for the entire influenza season and who had direct contract with patients and provided healthcare. 2,393 voluntarily vaccinated HCWs vs 215 unvaccinated HCWs after campaigns.<br><br>Exclusion: pregnancy, history of allergic reaction to flu vaccine or egg derivatives	- Influenza-like illness (ILI)<br>- Absenteeism due to ILI	ILI was defined as the occurrence of respiratory illness with at least one systematic symptom (fever, chills, myalgia) and at least one respiratory tract symptom (rhinorrhea, sore throat cough, hoarseness)
Chan (2007)	Hong Kong, 2004-2005<br>An emergency department of a general acute-care hospital<br>(Epi)	Retrospective cohort study<br><br>Vaccine: Trivalent inactivated vaccine	Full-time clinical staffs including nurses and healthcare attendants of the emergency department from November 2004 to October 2005 were included. 33 vaccine recipients were compared with 40 non-recipients.<br><br>Exclusion: Subjects with absence (not sickness related) from the department for longer than 1 month during the study period. Physicians were not also included.	- Absenteeism due to ILI	ILI included conditions features upper respiratory tract infection symptoms, fever, headache, or myalgia.
Chan (2008)	Taiwan, 2004-2005<br>Hospital<br>(Epi / Econ)	Retrospective cohort study<br><br>Vaccine: Trivalent vaccine	From the total workforce of 3,079 employees, 2,694 employees voluntarily received vaccines in a free vaccine program in the hospital. 447 employers in the selected department was provided questionnaires and of which 407 were further analyzed in the study. In the group, 367 employees were vaccinated and 40 were not vaccinated.<br><br>Exclusion: pregnant women, persons with a history of egg allergy or a previous allergic reactions to influenza vaccine.	- ILI<br>- Absenteeism<br>- Costs associated with ILI	ILI was defined as the incidence of at least 2 days duration with at least one of the following symptoms: fever, chills, myalgia, and at least one respiratory tract symptoms: rhinorrhea, sore throat, cough and hoarseness.
Colombo (2006)	Italy,<br>2002 -2003<br>Public healthcare facilities (Econ)	Retrospective cohort study<br><br>Vaccine: Unknown	107 voluntarily vaccinated employees were compared with 107 unvaccinated employees matched for age, sex, and job category, working in the healthcare unit.	- Cost-effectiveness of the vaccination program.	Influenza-like illness (ILI) as clinically diagnosed in accordance with WHO definition of influenza.
Ishikane (2016)	Japan,<br>2014-2015<br>A long-term care facility with non-acute care hospital<br>(Epi)	Retrospective cohort study<br><br>Vaccine: Trivalent	All HCWs in the facility were targeted in the study, of which 288 were vaccinated and 50 were unvaccinated.	- RIDTs positive cases.	Positive results of rapid influenza diagnostic tests using nasal specimens. (RIDTs).

Study	Study location, period, research	Study design	Study participants	Primary outcomes	Influenza/case definition
Ito (2005)	Japan,<br>2002-2003<br>A private hospital<br>(Epi/Econ)	Retrospective cohort study<br><br>Vaccine: One dose (0.5ml) of trivalent vaccine.	366 out of 370 HCWs invited to survey in May 2003 returned (98.9%). Of which, 237 received vaccines and 129 were unvaccinated.	- ILI case<br>- Absenteeism<br>- Cost effectiveness	Influenza was defined as self-reported febrile episodes with positive reaction on the rapid antigen detection test (RIDT) for influenza
Kheok (2008)	Singapore, 2004-2005<br>Two university and public hospitals<br>(Epi)	Prospective cohort study<br><br>Vaccine: unknown	All HCWs from the study hospitals were eligible. 541 agreed to participate the study, of which 211 were vaccinated.<br><br>Exclusion criteria: declining to give consent, history of egg protein allergy, neurological or immunological conditions that are contraindications to the influenza vaccine.	- ILI episodes<br>- Absenteeism due to sickness	ILI was defined as a respiratory illness of at least 2 days' duration consisting of at least one systematic symptom (fever, chills, myalgia) and at least one respiratory tract symptom (coryza, sore throat cough, hoarseness)
Michiels (2006)	Belgium,<br>2002-2003<br>GPs in Flanders, Belgium<br>(Epi)	Prospective cohort study<br><br>Vaccine: Intramuscular injection of 0.5 ml non-adjuvant trivalent inactivated split-influenza vaccine.<br>The same strains in vaccines were administered in 2002 and 2003.	GPs working in Flanders were recruited and vaccinated on a voluntary basis in July and August 2002 and 2003.<br><br>In the first year, 77 out of 122 GPs were initially vaccinated. In the second year, 100 out of 140 GPs received the vaccine. However, due to missing diaries and titers, the subjects included in analyses were 55 vaccinated and 33 unvaccinated GPs in 2002, and 30 vaccinated and 36 unvaccinated GPs in 2003.	- Respiratory tract infections<br>- RT-PCR positive cases<br>- Serologically-defined cases	A serologically confirmed influenza infections which is defined as a 4-fold or greater titer rise detected in the pair sample (pre- and post-epidemic).<br><br>RTI with and without positive RT-PCR tests<br><br>RTI with the positive results of nose and throat swabs with rapid test (RT-PCR)
Saxén (1999)	Finland, 1996-1997<br>Two pediatric hospitals<br>(Epi)	RCT<br><br>Vaccine: intramuscular injection of trivalent vaccine.<br>October 1996 to following April 1997	The study population was consisted of 800 eligible HCWs. These HCWs either did or did not have direct contact with patients. A total 547 out of 800 were randomized and immunized; 216 vaccinated and 211 control (placebo)<br><br>Exclusion criteria: Have an allergy to influenza vaccine. Those who have high risk of influenza infection and have been recommended to take the vaccine. Pregnancy was not excluded.	- Absenteeism due to respiratory infection	Respiratory illness and all sickness during the flu season. No viral or serological tests were conducted.

Study	Study location, period, research	Study design	Study participants	Primary outcomes	Influenza/case definition
Thomson (1999)	Australia, 1996 - 1997<br>One metropolitan hospital<br>(Epi / Eco)	Retrospective cohort study<br><br>Vaccine: inactivated vaccine	748 who participated the free vaccination program in 1997 was the vaccination group and the remainder of 3,844 hospital staffs was the control group.<br><br>Exclusion: Persons not on the hospital payroll database (e.g. volunteer work) were excluded.	- All-cause absenteeism<br>- Costs benefits of vaccination	All sickness
Van Buynder (2015)	Canada, 2012 - 2013<br>Public healthcare facilities in British Columbia<br>(Epi / Eco)	Retrospective cohort study<br><br>Vaccine: unknown	10,079 HCWs who worked in both pre-flu (1st January to 30th September in 2012) and flu periods (1st October 2012 to 28 February in 2013) were eligible; 2,360 were unvaccinated and 7,719 were vaccinated.<br><br>Exclusion criteria: volunteers and contracted staffs were excluded because there is no record of their absenteeism.	- Sick absenteeism	All sickness
Weingarten (1988)	U.S.,1985-1986<br>A hospital<br>(Epi / Eco)	RCT<br><br>Vaccine: 0.5mL dose of intramuscular injection of trivalent inactivated vaccine	179 full-time employees at the hospital between the ages of 21 and 65 years; 91 in the vaccinated group and 88 in the placebo group.<br><br>Exclusion criteria: to be pregnant, allergic to egg, have a history of adverse reaction or a chronic debilitating disease.	- ILI<br>- All-cause absenteeism<br>- Severity of illness scaled<br>- Adverse reactions to vaccines	ILI as defined by the CDC case definition of clinical influenza, which was a documented temperature greater than 100°F and at least the symptoms of cough or sore throat.
Wilde (1999)	U.S., 1992-1994<br>A university hospital in Baltimore<br>(Epi)	RCT<br><br>Vaccine: 0.5mL dose of intramuscular injection of trivalent inactivated vaccines.<br><br>Cases from October to following May were considered.	Study participants were hospital-based physicians, nurses, and respiratory therapists from departments of pediatrics, medicine, and emergency medicine. Total 264 HCWs during the 3 flu season study. 49 subjects participated for 2 seasons and 23 for 3 seasons.<br><br>Eligible subjects: < 50 year old.<br>Exclusion criteria: history of allergic reaction to influenza vaccine or egg products, allergy to the control vaccines, pregnancy, or medical conditions that would place the subject at high risk for complications from influenza infection	- Serologically-defined cases<br>- Respiratory illness<br>- Febrile respiratory illness<br>- Absenteeism	Influenza infection: a 4-fold increase in hemagglutination-inhibiting antibodies between the post immunization and post epidemic specimens.<br><br>Respiratory illness: report of 2 or more of the following symptoms for 2 or more days: rhinorrhea, cough, or sore throat.<br><br>Febrile respiratory illness was defined as respiratory illness with a report of fever.
