# Supplementary material for: A systematic review and meta-analysis of the direct epidemiological and economic effects of seasonal influenza vaccination on healthcare workers
Source: PLoS One. 2018 Jun 7;13(6):e0198685. doi: 10.1371/journal.pone.0198685 (PMC5991711; doi:10.1371/journal.pone.0198685)
Supplement: S3 Table — (PDF) [file pone.0198685.s005.pdf]

S3 Table. Epidemiological and economic outcomes

Study	Disease outcome	Absenteeism	Other effects/Adverse effects	Economic effects
Amadio (2010)	NA	The cases of absenteeism due to ILI was 7 / 215 (3.3%) among vaccinated compared to 171 / 2,393 (7.1%) among the unvaccinated The adjusted OR was 2.18 (95% CI:2.18-4.71).<br><br>The mean of the cumulative days of sick leave due to ILI among the unvaccinated was longer than the vaccinated, 4.7 days vs. 3.6 days (p< 0.001)	NA	NA
Chan (2007)	NA	The mean cumulative sick leave days per person was smaller in vaccine recipients (1.0 day) than in vaccine non-recipients (1.75 days) which was not significantly different (p=0.14). The proportion of subjects took sick leave among the vaccinated (30.3%) due to ILI was significantly larger than the unvaccinated group (55%).	NA	NA
Chan (2008)	50 out of 367 vaccinated and 6 out of 40 unvaccinated employees reported ILI, which the difference was not significant.	14 in vaccinated and 3 in unvaccinated took sick leave. The average number of work days loss due to ILI was 1.09 in vaccinated and 1.5 in unvaccinated groups.	32% (120/367) in the vaccinated group had local or systematic adverse events, the most frequent tiredness, myalgia, pain at injection site, headache, fever. Two employees had an allergic reaction such as itching and redness at the injection site.	The total costs of the vaccine program were US$4,278 including the direct cost of US$347 and indirect cost of US$3,931. The cost of vaccine was not borne by their hospital and therefore excluded from the calculation of total direct cost. The hospital may save US$21.5 per day of saved "lost workdays" per vaccinated employee.
Colombo (2006)	24 individuals of the vaccinated cohort and 31 individuals of the non-vaccinated cohort claimed to take sick leaves due to flu, and the difference was not significant (p=0.317)	The total working days lost due to flue was 161 and 231 days for the vaccinated and unvaccinated groups. The difference was not significant (p=0.329)	NA	Evaluations were based the employer's perspective, therefore costs sustained by employees such as medical tests, doctor visits and hospitalization were not considered.<br><br>The cost–benefit ratio showed the €1 investment of the vaccine program returns €4 through less absenteeism from work.
Ishikane (2016)	29/288 among the vaccinated and 7/50 among the unvaccinated had influenza infection - which RR was 0.72 (0.33-1.70) showing no difference between the groups.	NA	NA	NA

Study	Disease outcome	Absenteeism	Other effects	Economic effects
Ito (2005)	8/237 among vaccinated had influenza infections whereas 11/129 among unvaccinated had. The difference was statistically significant (p<0.01).<br><br>Among those who had influenza infections, the mean days of fever was 4.6 in the vaccinated and 4.9 in the unvaccinated (p=0.76).	Days of absent from work for the unvaccinated was significantly longer (0.04 days) than the vaccinated (0.15 days) (p < 0.001).<br><br>Among those who had influenza infections, the mean days of absent from work was 1.2 in the vaccinated and 1.8 in the unvaccinated (p=0.21).	20 vaccinated HCWs had adverse reactions; 19 were local reactions such as soreness at the vaccination site and 2 were general malaise. No serious adverse reactions occurred and no one was absent from work due to adverse reactions.	The absolute reduction of 11.1 days absent from work per 100 corresponds to a number of needed to treat of 10, to prevent 1 day absent from due to influenza. The cost of vaccination for 10 HCWs was 8,503 yen and the wage per day was 11,662 yen. Thus the vaccination cost was lower than the cost of 1 day's pay to an absent worker.
Kheok (2008)	132/211 vaccinated reported ILI compared to 182/330 unvaccinated - which RR of self-reported ILI was 1.13 (95%CI, 0.98-1.13; p=0.11). For matched vaccines, RR was 0.42 (95%CI, 0.37-0.66; p=<0.01) with VE 51% while unmatched vaccine RR was 1.22 (95%CI, 1.01-1.47; p=0.082) with VE -22%.	The mean medical leave was reduced for vaccinated group (0.26 ± 0.6 (SD) days in each visit) compared with non-vaccinated (0.3 ± 0.5) with p=0.406.<br><br>For matched vaccines, the mean medical leave was 0.1± 0.28 while it was 0.40± 0.93. The differences with unvaccinated was significant for matched vaccines (p < 0.01) though it was not for unmatched (p=0.42). However vaccines were unclear for several cases.	NA	NA
Michiels (2006)	There were no differences in the rates of RTI as well as RTI with positive swabs results. Serologically, there were also no differences between two groups in each year, but when combined two years, there were significant differences serologically.<br><br>In multivariate analyses, the interaction between age and vaccination was also found significant. When age was younger (30 years old), there was significant protective effects while there was no effects for the older groups, such as 50 years old.	NA	As the number of family number increases, the risk of influenza infections also significantly increases.	NA

Study	Disease outcome	Absenteeism	Other effects	Economic effects
Saxén (1999)	The episodes of respiratory infections (2.0 episodes of the controls vs. 1.8 of the vaccines) and the total number of days the study subjects suffered from respiratory infections (14.6 days of the controls vs. 13.5 of the vaccines) were not significantly different.	The vaccinated group had significantly fewer total sick leave days due to respiratory infections than control group (301 days vs 218 days; p=0.02)<br><br>The vaccinated group had also the fewer total number of days felt unable to work (3.5 days vs. 2.5 days, P = 0.02) than the unvaccinated group.	Antimicrobial prescription for the treatment of respiratory infections were not significantly different. Adverse effects: Local pain was significantly higher for the vaccinated group than the placebo group. Three vaccinees were absent from work for total 4 days due to the local pain.	NA
Thomson (1999)	NA	The group of immunization service participants had 2.41 hours/1,000 scheduled hours less for sick leaves than the non-participants group.<br><br>RR adjusting for staff category showed the immunized group had significantly less incidence of sick leaves (RR 0.92; 95%CI[0.86, 0.98]) while crude RR did not show any statistical differences (RR 1.02; 95%CI[0.96, 1.09]).	NA	Cost based on absenteeism was analyzed. With an assumption that the administration of a vaccine takes 0.75 hr for immunization, each staff saves 1.59 hrs in sick absence by immunization. Given the cost of vaccines and the average staff salaries, the benefit to the hospital would be between $1.55 and $5.80 /hr depending on salary level.
Van Buynder (2015)	NA	The mean differences in sick leave hours between pre-flu and flu season for vaccinated and vaccinated groups were 0.56hr/100 scheduled hours and 1.10 year/100 scheduled hours (p <0.001)<br><br>A linear regression showed an unvaccinated status had an increased absenteeism rate of of 0.5hr (95%CI: 0.2-0.9) /100 scheduled hours (p=0.004). In the regression, model age was included but did not change the estimate.	NA	Based on the estimated an excess of 3.3 sick hours for unvaccinated employees, the study estimates the cost saving of over $1.25m by vaccinated staff.

Study	Disease outcome	Absenteeism	Other effects	Economic effects
Weingarten (1988)	Vaccine did not significantly reduce the incidence of illness, duration of fever, or severity of illness. No difference in antipyretic usages in the two groups. However, among those who became ill with clinical influenza, there was a trend toward a shorter duration of illness among vaccinated persons.	There was no difference in absenteeism immediately after immunization and during the flu season.	Antipyretic usages: no difference between the two groups.<br><br>Adverse effects: experiences of erythema and pain was significantly higher in the vaccinated group, however no subject required medical attention due to an adverse reaction to the vaccine.	Vaccination cost was estimated based on vaccines, its equipment, and salary of a nurse to administrate immunization. There were no costs derived from absenteeism attributable to the vaccine or treatment of adverse reactions to the vaccine. No comparison analysis between the vaccinated and unvaccinated groups. Influenza immunization of hospital employees was performed at minimal cost and risk, but observed benefits were limited to a slight reduction in the duration of illness.
Wilde (1999)	Overall incidence of influenza infection from 1992 -1995 was 1.7% among vaccine recipients vs 13.9% among controls.<br><br>None of the vaccinated persons (n=3) who were serologically confirmed with infection did not report febrile respiratory symptoms. The unvaccinated persons who were serological evidence of infection A or B (n=24) were more likely than those without evidence to have more episodes (58% vs 14%; p <0.001) and longer duration (1.67 vs 0.20 days; p<0.001) of febrile respiratory illness.<br><br>The vaccinated group had fewer cumulative days of febrile respiratory illness than controls (28.7 days per 100 subjects vs 40.6 days per 100 subjects; p=0.57).	None of the vaccinated persons who were serologically confirmed with infection did not report work absence. The unvaccinated persons who were serological evidence of infection were more likely than those without evidence to have had more absenteeism (29% vs.7.7%; p=0.006) and higher number of days absent (0.67 vs. 0.14 days;p=0.001).<br><br>The vaccinated group also had fewer cumulative days of work absence than those in the control group (9.9 days per 100 subjects vs 21.1 days per 100 subjects; p = 0.41)	Adverse effects: there were no absences due to vaccine adverse effects during the observation period 3 days after vaccination. Other than mild pain or swelling at the injection site, the rest of the subjects reported no significant adverse effects.	NA
