# Supplementary material for: A systematic review and meta-analysis of the direct epidemiological and economic effects of seasonal influenza vaccination on healthcare workers
Source: PLoS One. 2018 Jun 7;13(6):e0198685. doi: 10.1371/journal.pone.0198685 (PMC5991711; doi:10.1371/journal.pone.0198685)
Supplement: S4 Table — (PDF) [file pone.0198685.s006.pdf]

S4 Table. The NOS score results of the observational studies

Study	SELECTION				COMPARABILITY	OUTCOME			Score	Quality
	Representativeness of exposed cohort	Selection of non-exposed cohort	Ascertainment of exposure	Outcome not present at the beginning of study	Comparability of cohorts	Assessment of outcome	Length of follow-up	Adequacy of follow-up		
Amadio	*	*	*	*	**	*	*	*	9	High
Chan(07)	*	*					*		3	Low
Chan (08)		*	*	*	*			*	5	Moderate
Colombo	*	*			**		*		5	Moderate
Ishikane	*	*		*	**	*	*		7	High
Ito	*	*		*	*		*		5	Moderate
Kheok	*	*	*		*				4	Low
Michiels		*		*	**	*	*		6	Moderate
Thomson	*	*			*	*	*		5	Moderate
Van Buynder	*	*	*	*	**	*	*	*	9	High
