# Supplementary material for: A systematic review and meta-analysis of the direct epidemiological and economic effects of seasonal influenza vaccination on healthcare workers
Source: PLoS One. 2018 Jun 7;13(6):e0198685. doi: 10.1371/journal.pone.0198685 (PMC5991711; doi:10.1371/journal.pone.0198685)

S1 Fig. Forest plot showing the vaccine effects on laboratory-confirmed cases.
The effects of seasonal influenza vaccines on the laboratory-confirmed cases between vaccinated and unvaccinated HCWs by study design.

Study or Subgroup	Vaccinated Events	Vaccinated Total	Unvaccinated Events	Unvaccinated Total	Weight	Risk Ratio IV, Random, 95% CI
**1.2.1 Non-randomized studies**						
Ishikane 2016	29	288	7	50	23.4%	0.72 [0.33, 1.55]
Ito 2006	8	237	11	129	20.3%	0.40 [0.16, 0.96]
Michiels 2006	5	95	13	69	18.0%	0.28 [0.10, 0.75]
Michiels 2006	14	162	11	75	24.2%	0.59 [0.28, 1.24]
**Subtotal (95% CI)**		**782**		**323**	**85.8%**	**0.50 [0.33, 0.76]**
Total events	56		42			
Heterogeneity: Tau² = 0.00; Chi² = 2.66, df = 3 (P = 0.45); I² = 0%						
Test for overall effect: Z = 3.27 (P = 0.001)						
**1.2.4 Randomized studies**						
Wilde 1999	3	180	24	179	14.2%	0.12 [0.04, 0.41]
**Subtotal (95% CI)**		**180**		**179**	**14.2%**	**0.12 [0.04, 0.41]**
Total events	3		24			
Heterogeneity: Not applicable						
Test for overall effect: Z = 3.46 (P = 0.0005)						
**Total (95% CI)**		**962**		**502**	**100.0%**	**0.40 [0.23, 0.69]**
Total events	59		66			
Heterogeneity: Tau² = 0.17; Chi² = 7.42, df = 4 (P = 0.12); I² = 46%						
Test for overall effect: Z = 3.32 (P = 0.0009)						
Test for subgroup differences: Chi² = 4.76, df = 1 (P = 0.03), I² = 79.0%						

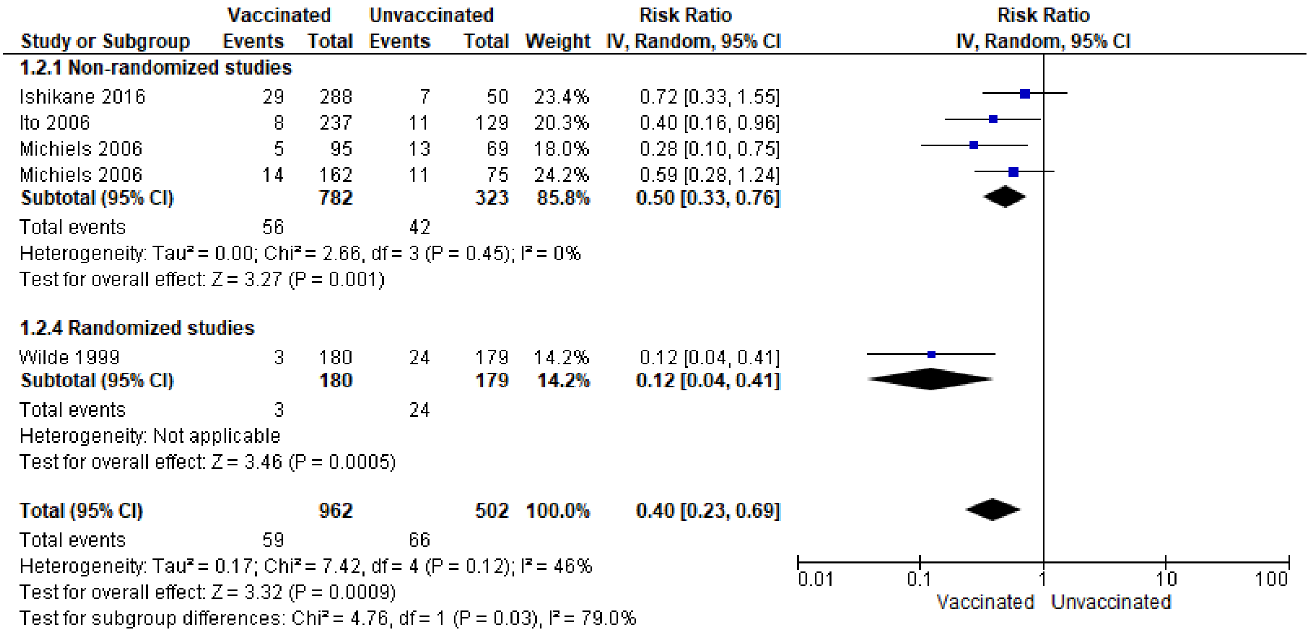

Supplement: S1 Fig — The effects of seasonal influenza vaccines on the laboratory-confirmed cases between vaccinated and unvaccinated HCWs by study design. (PDF) [file pone.0198685.s007.pdf]
