# Supplementary material for: A systematic review and meta-analysis of the direct epidemiological and economic effects of seasonal influenza vaccination on healthcare workers
Source: PLoS One. 2018 Jun 7;13(6):e0198685. doi: 10.1371/journal.pone.0198685 (PMC5991711; doi:10.1371/journal.pone.0198685)

S2 Fig. Forest plot showing the vaccine effects on absenteeism incidence.
The effects of seasonal influenza vaccines on the absenteeism incidence between vaccinated and unvaccinated HCWs by study design.

	Vaccinated		Unvaccinted			Risk Ratio
**Study or Subgroup**	**Events**	**Total**	**Events**	**Total**	**Weight**	**IV, Random, 95% CI**
**2.3.1 Non-randomized studies**						
Amodio 2010	7	215	171	2393	6.3%	0.46 [0.22, 0.96]
Chan 2007	10	33	22	40	9.4%	0.55 [0.31, 0.99]
Chan 2008	14	367	3	40	2.6%	0.51 [0.15, 1.69]
Colombo 2006	24	107	31	107	13.7%	0.77 [0.49, 1.23]
Thomson 1999	424	748	2368	3844	47.4%	0.92 [0.86, 0.98]
**Subtotal (95% CI)**		**1470**		**6424**	**79.4%**	**0.73 [0.55, 0.98]**
Total events	479		2595			
Heterogeneity: Tau² = 0.05; Chi² = 7.57, df = 4 (P = 0.11); I² = 47%						
Test for overall effect: Z = 2.12 (P = 0.03)						
**2.3.2 Randomized studies**						
Weingarten 1988	39	91	38	88	20.6%	0.99 [0.71, 1.39]
**Subtotal (95% CI)**		**91**		**88**	**20.6%**	**0.99 [0.71, 1.39]**
Total events	39		38			
Heterogeneity: Not applicable						
Test for overall effect: Z = 0.04 (P = 0.97)						
**Total (95% CI)**		**1561**		**6512**	**100.0%**	**0.82 [0.67, 1.00]**
Total events	518		2633			
Heterogeneity: Tau² = 0.02; Chi² = 7.85, df = 5 (P = 0.16); I² = 36%						
Test for overall effect: Z = 1.95 (P = 0.05)						
Test for subgroup differences: Chi² = 1.83, df = 1 (P = 0.18), I² = 45.4%						

Risk Ratio
IV, Random, 95% CI

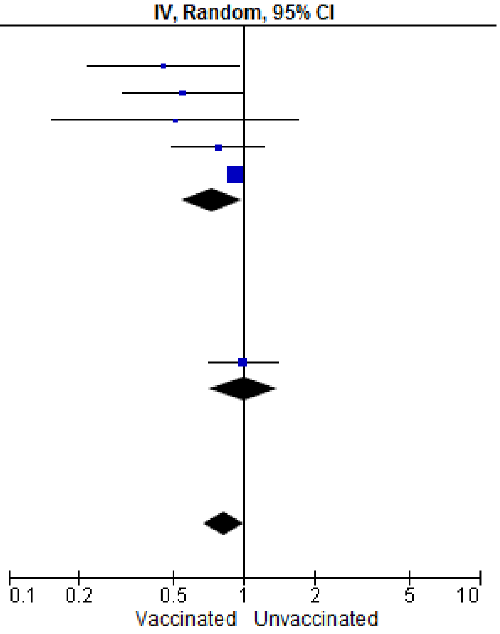

Supplement: S2 Fig — The effects of seasonal influenza vaccines on the absenteeism incidence between vaccinated and unvaccinated HCWs by study design. (PDF) [file pone.0198685.s008.pdf]
