# Supplementary figures and images for: A systematic review and meta-analysis of the direct epidemiological and economic effects of seasonal influenza vaccination on healthcare workers
Source: PLoS One. 2018 Jun 7;13(6):e0198685. doi: 10.1371/journal.pone.0198685 (PMC5991711; doi:10.1371/journal.pone.0198685)

**S3 Fig. The sensitivity analysis for ILI incidence after removing studies with low quality.**

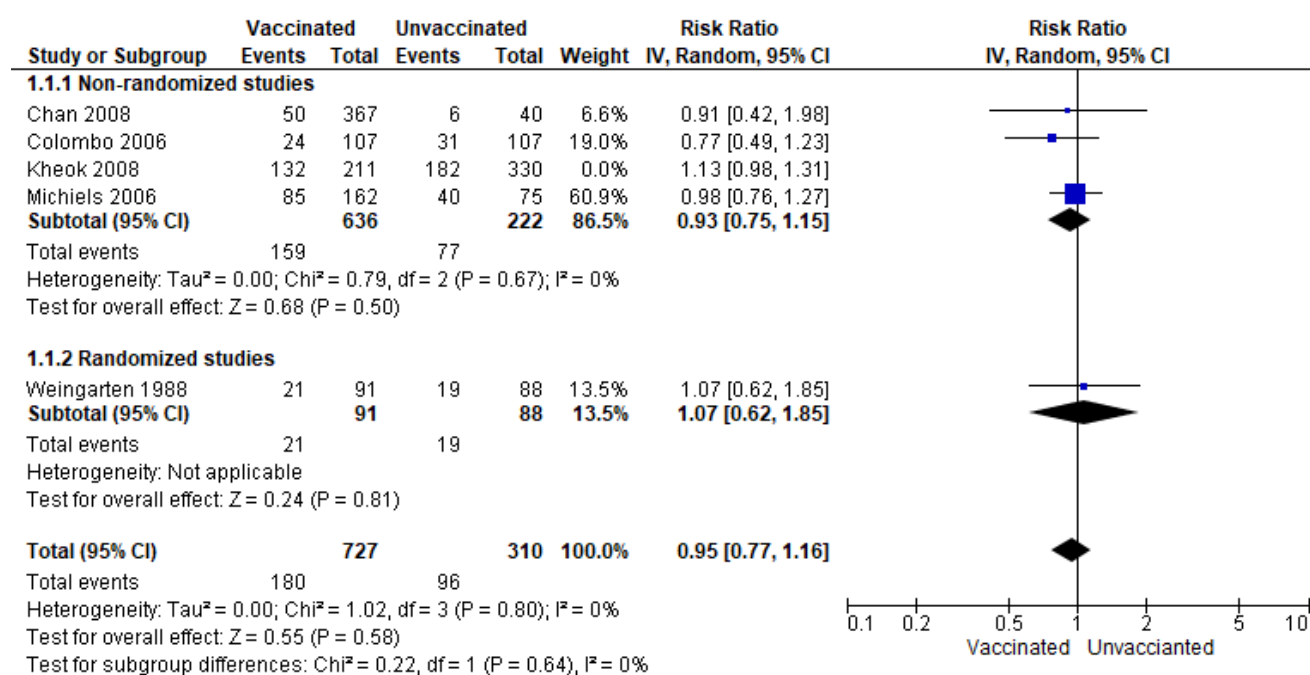

Supplement: S3 Fig — (PDF) [file pone.0198685.s009.pdf]
