# Supplementary material for: A systematic review and meta-analysis of the direct epidemiological and economic effects of seasonal influenza vaccination on healthcare workers
Source: PLoS One. 2018 Jun 7;13(6):e0198685. doi: 10.1371/journal.pone.0198685 (PMC5991711; doi:10.1371/journal.pone.0198685)

S4 Fig. The sensitivity analysis for absenteeism incidence after removing studies with low quality.

Study or Subgroup	Vaccinated Events	Vaccinated Total	Unvaccinted Events	Unvaccinted Total	Weight	Risk Ratio IV, Random, 95% CI
**2.4.1 ILI**						
Amodio 2010	7	215	171	2393	4.4%	0.46 [0.22, 0.96]
Chan 2007	10	33	22	40	0.0%	0.55 [0.31, 0.99]
Chan 2008	14	367	3	40	1.7%	0.51 [0.15, 1.69]
Colombo 2006	24	107	31	107	10.4%	0.77 [0.49, 1.23]
**Subtotal (95% CI)**		**689**		**2540**	**16.6%**	**0.65 [0.45, 0.94]**
Total events	45		205			
Heterogeneity: Tau² = 0.00; Chi² = 1.59, df = 2 (P = 0.45); I² = 0%						
Test for overall effect: Z = 2.26 (P = 0.02)						
**2.4.2 All-cause sickness**						
Thomson 1999	424	748	2368	3844	66.0%	0.92 [0.86, 0.98]
Weingarten 1988	39	91	38	88	17.4%	0.99 [0.71, 1.39]
**Subtotal (95% CI)**		**839**		**3932**	**83.4%**	**0.92 [0.86, 0.99]**
Total events	463		2406			
Heterogeneity: Tau² = 0.00; Chi² = 0.19, df = 1 (P = 0.67); I² = 0%						
Test for overall effect: Z = 2.38 (P = 0.02)						
**Total (95% CI)**		**1528**		**6472**	**100.0%**	**0.88 [0.75, 1.03]**
Total events	508		2611			
Heterogeneity: Tau² = 0.01; Chi² = 5.05, df = 4 (P = 0.28); I² = 21%						
Test for overall effect: Z = 1.58 (P = 0.11)						
Test for subgroup differences: Chi² = 3.27, df = 1 (P = 0.07), I² = 69.4%						

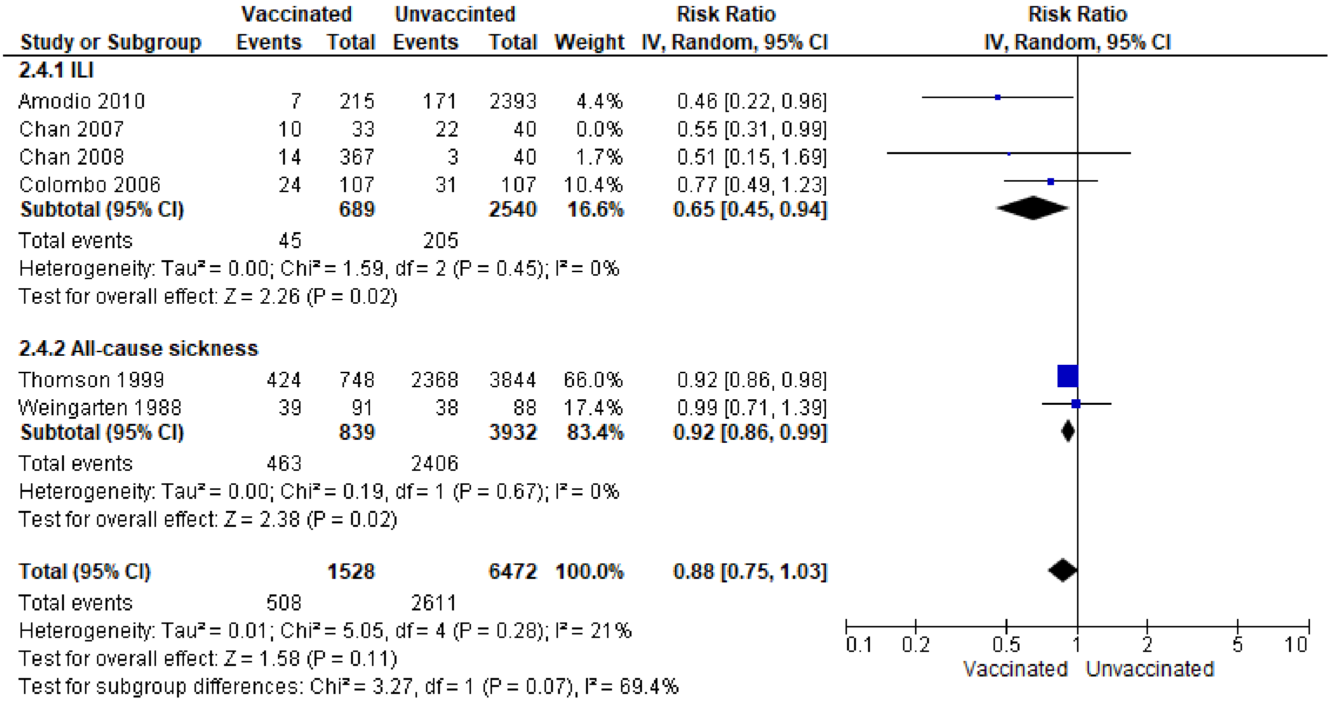

Supplement: S4 Fig — (PDF) [file pone.0198685.s010.pdf]
