# Supplementary material for: A systematic review and meta-analysis of the direct epidemiological and economic effects of seasonal influenza vaccination on healthcare workers
Source: PLoS One. 2018 Jun 7;13(6):e0198685. doi: 10.1371/journal.pone.0198685 (PMC5991711; doi:10.1371/journal.pone.0198685)

S5 Fig. The sensitivity analysis for the mean days of absenteeism after removing studies with low quality.

	Vaccinted			Unvaccinted				Mean Difference
Study or Subgroup	Mean	SD	Total	Mean	SD	Total	Weight	IV, Random, 95% CI
**3.2.1 ILI**								
Amodio 2010	3.6	1.2105	7	4.7	1.2105	171	8.8%	-1.10 [-2.01, -0.19]
Chan 2007	3.3	2.79	10	3.18	1.94	22	0.0%	0.12 [-1.79, 2.03]
Chan 2008	1.09	0.507	14	1.5	0.01	3	88.7%	-0.41 [-0.68, -0.14]
**Subtotal (95% CI)**			**21**			**174**	**97.5%**	**-0.61 [-1.22, 0.00]**
Heterogeneity: Tau² = 0.12; Chi² = 2.01, df = 1 (P = 0.16); I² = 50%								
Test for overall effect: Z = 1.95 (P = 0.05)								
**3.2.2 Lab-confirmed**								
Ito 2006	1.2	1.8902	8	1.8	1.8902	11	2.5%	-0.60 [-2.32, 1.12]
**Subtotal (95% CI)**			**8**			**11**	**2.5%**	**-0.60 [-2.32, 1.12]**
Heterogeneity: Not applicable								
Test for overall effect: Z = 0.68 (P = 0.49)								
**Total (95% CI)**			**29**			**185**	**100.0%**	**-0.48 [-0.75, -0.20]**
Heterogeneity: Tau² = 0.00; Chi² = 2.04, df = 2 (P = 0.36); I² = 2%								
Test for overall effect: Z = 3.41 (P = 0.0007)								
Test for subgroup differences: Chi² = 0.00, df = 1 (P = 0.99), I² = 0%								

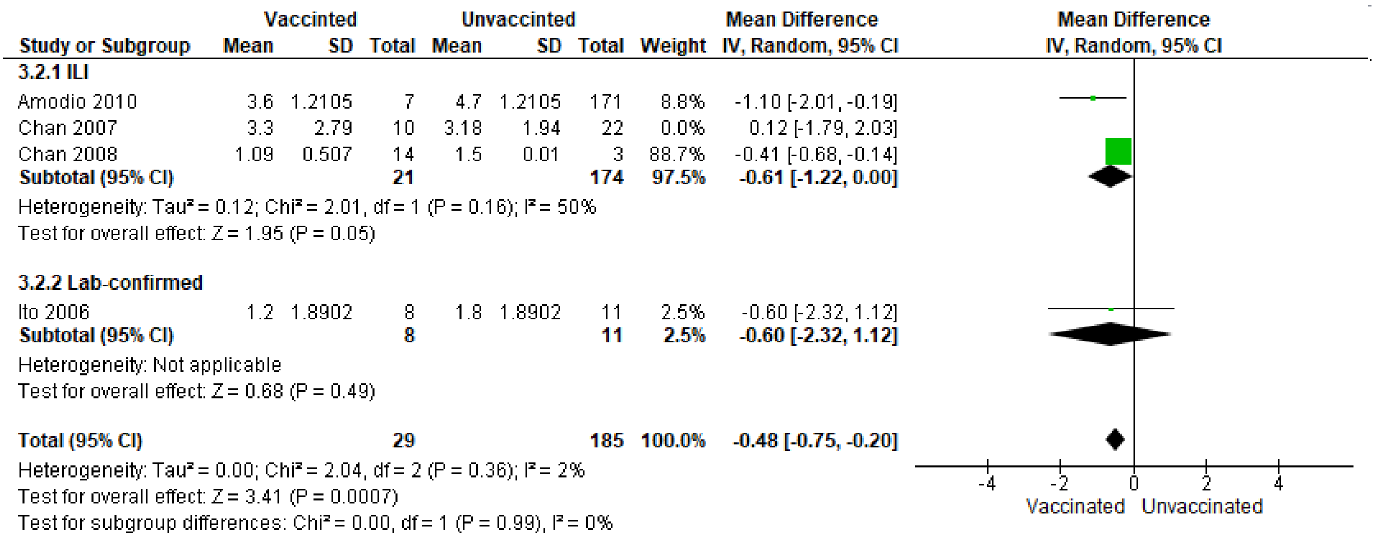

Supplement: S5 Fig — (PDF) [file pone.0198685.s011.pdf]
